# Macular inner retinal layers in multiple sclerosis

**DOI:** 10.3389/fneur.2025.1549091

**Published:** 2025-03-31

**Authors:** Ori Zahavi, Maria Nilsson, Ali Manouchehrinia, Rune Brautaset, Ingrid Kockum, Abinaya P. Venkataraman, Alberto Dominguez-Vicent

**Affiliations:** ^1^Unit of Optometry, Division of Eye and Vision, Department of Clinical Neuroscience, Karolinska Institute, Stockholm, Sweden; ^2^Department of Clinical Neuroscience, Karolinska Institutet, Stockholm, Sweden; ^3^Centrum for Molecular Medicine, Karolinska University Hospital, Stockholm, Sweden

**Keywords:** multiple sclerosis, optical coherence tomography, inner retinal layers, retinal nerve fiber layer, ganglion cell-inner plexiform layer

## Abstract

**Aim:**

To evaluate the structural changes of individual inner retinal layers in the macular area and identify the most affected layer in subgroups of multiple sclerosis (MS) patients compared to healthy controls (HC).

**Methods:**

In total, Optical coherence tomography (OCT) data from 507 MS patients and 183 HC were exported retrospectively. The MS patients were grouped according to MS sub-types, primary progressive (PP), Relapsing–Remitting (RR) and Secondary progressive (SP). Thickness of four inner retinal layers, the macula nerve fiber layer (NFL), ganglion cell layer (GCL), inner plexiform layer (IPL) and the inner nuclear layer (INL) were evaluated in nine sectors based on the Early Treatment Diabetic Retinopathy Study (ETDRS) map. The individual layer thickness measurements were compared between each MS subtype and HC while controlling for the potential confounding effects of age, sex, and previous history of ON.

**Results:**

The NFL was thinner in all inferior, superior, and nasal sectors in all MS subgroups. The thinning was more pronounced in the PP and SP groups. The thinning varied between 3 to 20% compared to HC. The GCL was also thinner, especially in the inner sectors of the ETDRS grid. The SP subgroup had the largest reduction (27.8%) in the inner nasal sector. The IPL was also reduced in all MS subgroups. In contrast to PP and SP groups, the RR group showed an increased INL thickness compared to HC in the inner sectors.

**Conclusion:**

Macular region is suitable for monitoring the neurodegeneration in MS. The macular NFL seems to have the strongest association with MS disease and may serve as a marker for global atrophy. The pattern of IPL reduction tends to follow the GCL, so these layers can be measured combined.

## 1 Introduction

Optical coherence tomography (OCT) has been recognized for acquiring non-invasive and high-resolution retinal images (1–3). It has emerged as a safe, fast, and non-invasive technique that can obtain cross-sectional images of the macula and optic nerve. With OCT, it is possible to obtain both qualitative and quantitative information on the retinal structures (4, 5).

The retina is part of the central nervous system (CNS), and the ganglion cell axons in the eye are un-myelinated. The ganglion cells and their axons can be measured and used to describe the overall neural degeneration associated with neurodegenerative diseases such as multiple sclerosis (MS). MS is a chronic, autoimmune, inflammatory demyelinating disease of the CNS that results in axonal and neuronal degeneration due to retrograde transsynaptic degeneration (6, 7).

In MS, measures of the peripapillary retinal nerve fiber layer (pRNFL) and the macular ganglion cell complex [ganglion cell + inner plexiform layer (GCIP)] have been proved to be sensitive parameters (8, 11). In a previous study, we found an association between thinner pRNFL and GCIP thicknesses and cognitive impairment as well as physical disability in a large cohort of MS. More specifically, the OCT parameter with the strongest association was the temporal portion of pRNFL. The pRNFL and macular GCIP could also differentiate MS subtypes, i.e., relapsing–remitting (RR), secondary progressive (SP), and primary progressive (PP) (12). The inner nuclear layer (INL) has been seen to increase in volume in MS patients with optic neuritis (ON) and the occurrence of clinical relapses (13). In contrast, in PPMS patients, the treatment is shown to prevent pathological thinning of the INL (14). The individual macular layers considered relevant in MS are the inner retinal layers: the nerve fiber layer (NFL), GCIP, and INL. Recent developments in the OCT software make it possible to identify and measure each retinal layer thickness separately and more reliably (15, 16).

The aim of the present study is to evaluate the structural loss of individual inner retinal layers and identify the most affected layer in patients with MS within the three subgroups: PPMS, RRMS and SPMS, and compare this with the healthy control group.

## 2 Methods and materials

### 2.1 Subjects

OCT data was collected retrospectively from a previous cross-sectional study (17), where MS patients were recruited consecutively during regular follow-up visits between May 2013 and October 2015 at the MS clinic of the Neurology Department (Karolinska University Hospital, Solna, Sweden). Subjects with a history of ocular disease other than ON, trauma, or systemic (eye) disease besides MS were excluded. Subjects with history of recent ON (within the last 6 months) were excluded. In total, data from examinations of 507 MS patients were exported retrospectively. MS subjects were grouped according to MS sub-types (PP, RR, SP, and history of ON). 183 healthy controls (HC) recruited from the Optometry clinic at Karolinska Institute were also included in the study. The study adhered to the tenets of the Declaration of Helsinki and was approved by the regional ethical committee. Written informed consent was obtained from the study participants.

### 2.2 Optical coherence tomography

**Figure 1:**
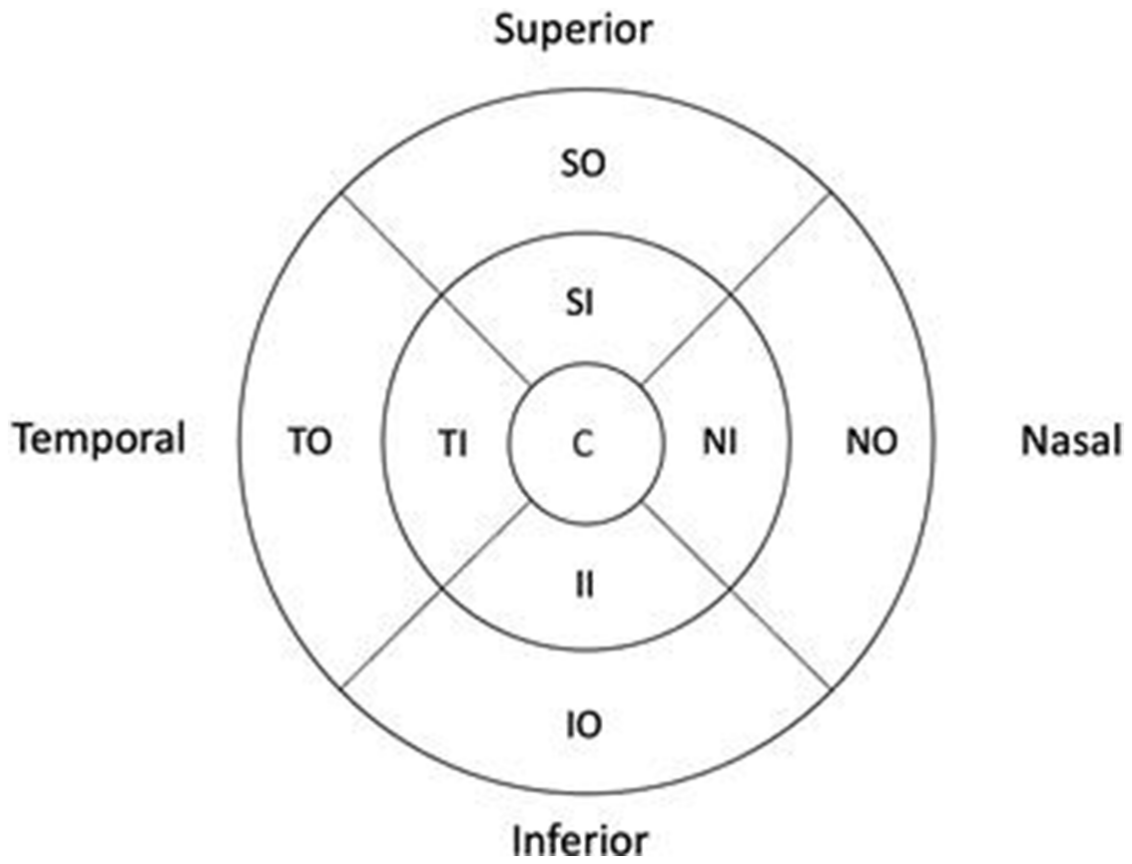
Early treatment diabetic retinopathy study macular map. C, Central; II, Inferior Inner; IO, Inferior Outer; NI, Nasal Inner; NO, Nasal Outer; SI, Superior Inner; SO, Superior Outer; TI, Temporal Inner; TO, Temporal Outer. All values were converted.

All subjects were examined with the Canon spectral domain OCT-HS100 (Tokyo, Japan). The OCT has a scanning speed of 70,000 A scans/s and an axial resolution of 3 μm. The 10 mm x 10 mm macular region scan is constructed from 128 vertically oriented B-scans, each consisting of 1,024 A-scans. The updated incorporated algorithm (Version 4.5) allowed the measurement of the 10 individual retinal layer thicknesses. For this study, we evaluated the four inner retinal layers: NFL, GCL, IPL, and INL. Each layer thickness in the macula area was exported from the ETDRS (Early Treatment Diabetic Retinopathy Study) thickness map (Figure 1), which consisted of nine sectors: central (C), inferior inner (II), inferior outer (IO), nasal inner (NI), nasal outer (NO), superior inner (SI), superior outer (SO), temporal inner (TI) and temporal outer (TO). The diameter of the central sector is 1 mm, the inner sector 3 mm, and the outer sector 6 mm. Measurements were obtained from both eyes of each subject in both groups. We only included scans without any artifacts and with a signal strength of ≥7 (maximum obtainable signal strength was 10) and scans in agreement with the OSCAR-1B criteria for further analysis.

### 2.3 Statistical analysis

R version 3.3.3 (26) and package geepack were used for data analyses. Generalized estimating equations models were used to account for within-patient inter-eye correlations. All 4 retinal layers in each of the 9 sectors were compared between each MS subtype and HCs while controlling for the potential confounding effects of age, sex, and previous history of ON.

## 3 Results

In total, the individual inner retinal layer thickness of 183 HCs and 507 MS patients were analyzed. Table 1 shows the demographic data of each MS sub-group and HC.

**Table 1:** Demographic overview of the study cohort.

	HC (*n* = 183)	RR (*n* = 358)	SP (*n* = 132)	PP (*n* = 17)
Mean age in years±SD	45.5 ± 15.7	39.4 ± 9.5	54.3 ± 10.6	53.8 ± 12.9
Females (%)	133 (72.7%)	266 (74.3%)	87 (65.9%)	8 (47.1%)
Mean disease duration in years±SD	NA	9.5 ± 6.9	22.2 ± 10.9	10.9 ± 6.7
History of ON (%)	NA	44 (12.3%)	20 (15.2%)	0

*n, number of subjects; HC, healthy controls; RR, relapsing–remitting multiple sclerosis; SP, secondary progressive multiple sclerosis; PP, primary progressive multiple sclerosis; SD, Standard deviation; NA, Not applicable; ON, Optic neuritis.*

**Figure 2:**
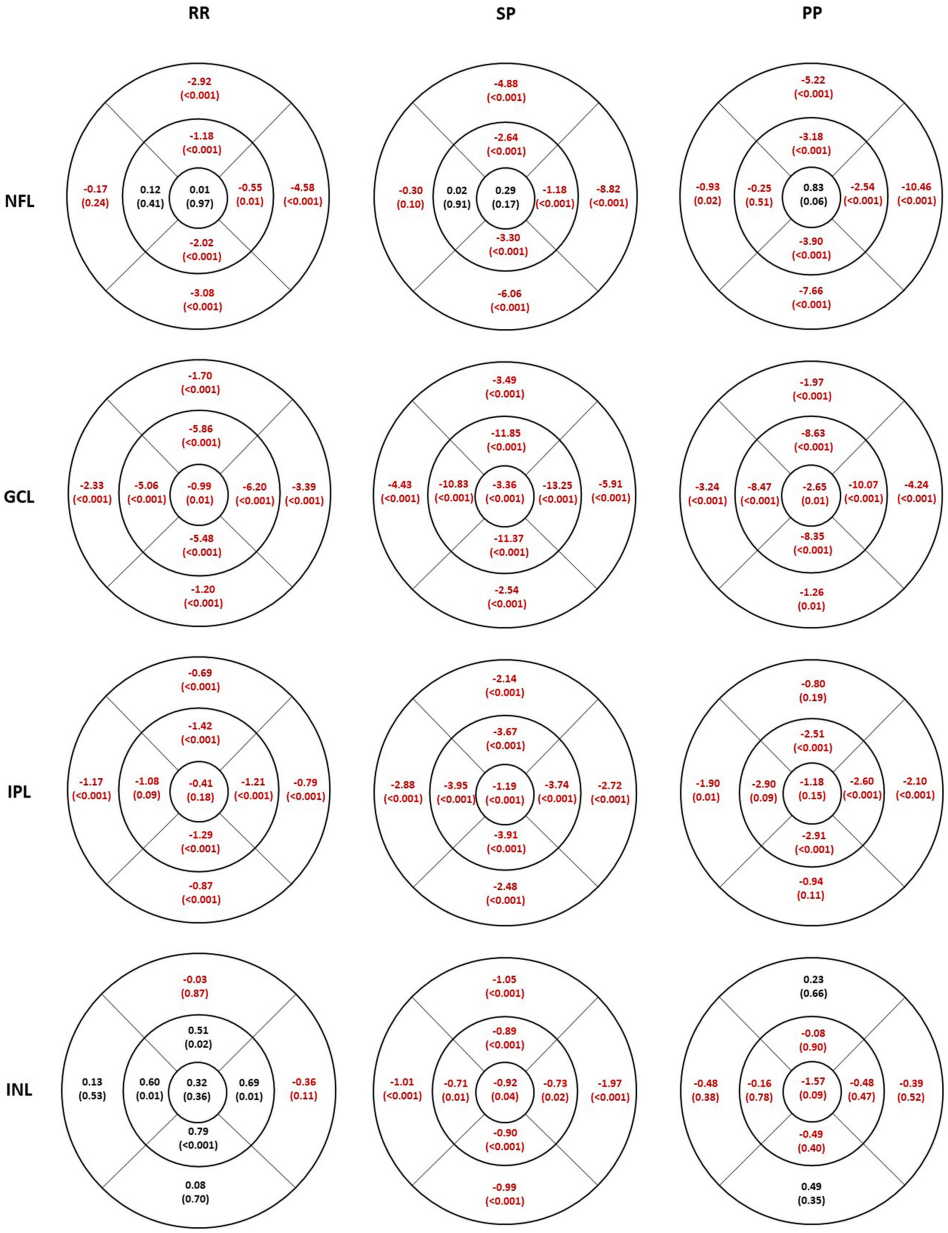
Individual inner retinal layer thickness differences in 3 multiple sclerosis sub groups compared to healthy controls. The values represent mean difference and the level of significance is given in parentheses. Thicker values are denoted in black and thinner values in red. RR, relapsing–remitting multiple sclerosis; SP, secondary progressive multiple sclerosis; PP, primary progressive multiple sclerosis; NFL, nerve fiber layer; GCL, ganglion cell layer; IPL, inner plexiform layer; INL, inner nuclear layer.

Figure 2 shows the mean difference in thickness values between HC and MS subgroups for each inner retinal layer. Sectors with a statistically significant thickness difference are marked in red (*p*-value included in parenthesis). Table 2 shows the mean thickness for each layer for HC and the percentage change in thickness in each MS subgroup compared to HC. Supplementary Table S1 shows the limits of 5–95% confidence interval for the thickness differences. In both Figure 2 and Table 2, a negative value indicates a decrease in thickness compared to HC.

**Table 2:** The mean retinal layer thickness in each sector for the healthy control group and the corresponding difference in percentage for the MS subgroups.

	Nerve fiber layer (values expressed in μm)				Ganglion cell layer (values expressed in μm)				Inner plexiform layer (values expressed in μm)				Inner nuclear layer (values expressed in μm)			
Sector	Mean (SD)	Diff. in %			Mean (SD)	Diff. in %			Mean (SD)	Diff. in %			Mean (SD)	Diff. in %		
	HC	RR	SP	PP	HC	RR	SP	PP	HC	RR	SP	PP	HC	RR	SP	PP
C	9.2 (2.5)	+0.5	+4.9	+10.4	17.3 (5.5)	−7.5	−20.2	−11.0	17 (4.6)	−3.5	−7.1	−3.5	18.5 (5.3)	+1.1	−2.2	−1.6
II	26.4 (3.2)	−8.7	−5.6	−13.3	50.1 (5.0)	−13.6	−25.1	−16.4	41 (3.5)	−3.9	−10	−5.6	43.1 (2.9)	+1.4	−2.3	−0.2
IO	40.1 (6.1)	−9.5	−17.5	−20	27.5 (2.6)	−4.4	−10.9	−5.5	32 (3.1)	−2.2	−10.3	−4	32 (2.6)	+0.9	−4.7	+0.6
NI	20.6 (2.5)	−3.4	−5.8	−10.7	51.5 (5.2)	−14	−27.8	−18.8	40.3 (3.5)	−4.5	−10.4	−5.2	43.2 (3.2)	+1.4	−1.6	−0.7
NO	48.2 (7.1)	−10.8	−19.9	−21.4	33.9 (3.6)	−10.6	−20.1	−13.9	34.5 (3.6)	−2	−10.4	−7	35.7 (2.9)	−0.6	−7.3	−2
SI	24.4 (3.0)	−5.3	−11.5	−11.5	50.8 (5.1)	−13.4	−25.4	−16.1	40.1 (3.3)	−4	−9.5	−4.7	43.1 (2.8)	+0.5	−2.6	−0.7
SO	37.5 (5.3)	−9.3	−15.2	−14.9	29.8 (2.9)	−6.7	−13.1	−6.7	33.1 (3.1)	−2.1	−9.1	−3.6	33.6 (2.7)	−0.9	−4.5	+0
TI	16.8 (2.2)	+0	+2.4	1.2	47.9 (4.6)	−13.4	−24.6	−16.3	38.8 (3.8)	−3.7	−10.3	−4.9	40.4 (3.1)	+0.5	−2.0	−0.7
TO	18.3 (1.7)	−2.2	−0	2.7	31.7 (3.7)	−8.5	−15.5	−9.8	35.4 (3.5)	−3.1	−10.5	−5.4	35.1 (2.8)	+0.9	−4.0	−1.1

HC, healthy controls; RR, relapsing–remitting multiple sclerosis; SP, secondary progressive multiple sclerosis; PP: primary progressive multiple sclerosis, SD, Standard deviation; C, Central; II, Inferior Inner; IO, Inferior Outer; NI, Nasal Inner; NO, Nasal Outer; SI, Superior Inner; SO, Superior Outer; TI, Temporal Inner; TO, Temporal Outer. Diff. in %, difference from HC in percentage.

The NFL was thinner in all inferior, superior, and nasal sectors in all MS subgroups (Figure 2, first row). The thinning was least pronounced in the RR group, followed by SP and PP. The temporal sector was not significantly different compared to the HC except for less than 1 micron in the outer sector in the PP group. The percentage difference in the inner sectors (inferior, superior, and nasal) is smaller than in the outer sector for all MS subgroups. The maximum thinning was seen in the outer inferior and nasal sectors.

The GCL (Figure 2, second row) was thinner in all sectors in all MS subgroups. The thinning and the percentage difference were more extensive in the inner sectors compared to the corresponding outer sectors. The thinning was more pronounced in the SP group.

The thinning in the IPL thickness (Figure 2, third row) was similar between the corresponding inner and outer sectors in all subgroups. The largest percentage decrease was seen in the SP subgroup, which had significantly thinner IPL thickness in all sectors. Similar to GCL, IPL was also thinner in all sectors; however, not all sectors were significantly different in the RR and PP subgroups compared to the HC.

The INL (Figure 2, fourth row) was significantly thinner in all sectors in the SP subgroup, whereas none were significantly different in the PP subgroup. In contrast, the RR subgroup showed an increased INL thickness compared to HC in the inner sectors, but no difference was seen in the other sectors.

## 4 Discussion

The aim of the present study was to evaluate the structural loss of individual inner retinal layers in the macula area and identify the most affected layer in subgroups of MS patients [relapsing–remitting (RR), secondary progressive (SP), and primary progressive (PP)] compared to controls. Results from the large cohort studied showed that NFL and GCL were reduced in all subgroups and in most sectors. The loss in IPL tended to follow GCL thinning, whereas the INL showed diverse variations in the different MS subgroups.

In our study, the NFL in the macula region was most affected in the nasal sector, followed by the inferior and superior sectors in all MS subgroups. The differences were more prominent in the outer compared to the inner sectors. The axons from the macular area related to the temporal portion of the ONH have their major contribution from the nasal sector. This finding is, therefore, in line with our previous studies showing the temporal pRNFL to be a sensitive parameter to MS-related retinal degeneration (17).

It is well known that normal aging is related to neuro-retinal thinning. The rate of thinning has been described as discrete before the 7th decade, so we believe the age difference between the groups in our cohort is not a confounding factor. The differences seen between MS subgroups indicate that the thinning is related to disease severity. This is supported by our previous results from the same MS cohort, showing similar EDSS and SDMT scores in the SP and PP groups, which were worse than the RR group (12). GCL thickness is a robust parameter describing MS-related atrophy, as all subgroups showed thinning in all sectors. The GCIPL thinning is shown to reflect the changes in the gray matter (GM) in MS (18, 19). Another factor we did not account for is the refractive error or correction for magnification effects due to different axial lengths. High refractive error may influence retinal thickness, resulting in a significantly thinner retina compared to subjects with average refractive error and the normative database is not available for higher refractive errors. However, due to the large sample size in the present study, we believe that this limitation would not influence the findings and the differences we see between groups.

Inner GCL sectors had the largest proportional thinning compared to the corresponding outer sectors in all MS subgroups. According to normal anatomy, GCL is always thickest in this area and corresponds to the foveal wall. The SP group showed the most thinning, followed by the PP and RR groups. It can be noted that the SP group had the longest disease duration, whereas the PP and RR groups had similar disease duration. The thinning pattern between groups was a bit different from the pattern of NFL thinning between groups. IPL followed the same trend as the GCL, with the SP group showing the maximum thinning. From these findings, we can hypothesize that we can measure these layers together without reducing the sensitivity needed to detect neuro-retinal degeneration. Measuring these layers together has been shown to be more precise than measuring these layers individually and the reasons could be that the automated segmentation might not be able to accurately delineate these two layers (20, 21).

When observing the INL, it was slightly thicker in the RR group compared to the HC group. However, the pattern of INL alteration was different in the other MS subgroups, with the SP group showing a significant discrete thinning and the PP group showing a thinning, which was not significantly different from the HC group. In any case, the variations in INL were minimal and were less than 2 microns. INL thickness in MS has been studied in the past. Though some studies suggested that the thickening of INL is due to microcystic macular edema or recent/acute ON, more recent studies indicate that the INL thickening can be present even without recent or acute ON (22–25). The inflammation process is most intense early in the disease course. The INL thickening seen in the RR group and the thinning seen in the SP group in our sample could, therefore, reflect the stage of the inflammatory or degeneration process. It should be noted that none of the subjects included in this study had any recent history of ON (in the last 6 months before the OCT measurements); the INL thickening seen in the RR group is not likely to be associated with ON. A common limitation in studies including the PP subgroup is the smaller sample size. The existence of ON history might reduce the sensitivity to use retinal layer thinning as a measure of global brain atrophy, therefore we considered this factor in the analysis. In any case, the use of OCT is still relevant to study progressive axon loss related to MS disease.

While comparing the values for GCL and IPL individually between the MS ON eyes and MS non-ON eyes with the healthy controls, we can see that the values differ by about 30 and 14% for GCL and 12 and 4% for IPL in ON eyes and non-ON eyes, respectively. If we compare the same but for combined GCL + IPL thickness, the values differ by 22 and 10% in ON eyes and non-ON eyes, respectively. Overall, it seems that measuring the inner retinal layer thickness individually or combined shows the same trend in the differences between the MS subgroups irrespective of ON history. This could also mean that the association between individual layer thickness and cognitive and physical functions would be similar to that seen with ganglion cell complex thickness. As the pattern of IPL reduction tends to follow the GCL and considering the fact that measuring the layers individually could be subjected to segmentation errors it could be acceptable and possibly more robust to measure the GCL + IPL complex.

## 5 Conclusion

Our results show that the macular region is suitable for monitoring the individual thickness layer of the retina in MS. The macular NFL seems to have the strongest association with MS disease phenotype and was most affected in the PP group, followed by the SP group and therefore could serve as a marker for global atrophy. GCL and IPL were also reduced in all MS subgroups and reflect the disease severity. As the pattern of IPL reduction tends to follow the GCL, these layers can also be measured combined.

## Data Availability

The raw data supporting the conclusions of this article will be made available by the authors, without undue reservation.
