# Supplementary material for: Macular inner retinal layers in multiple sclerosis
Source: Front Neurol. 2025 Mar 31;16:1549091. doi: 10.3389/fneur.2025.1549091 (PMC11997350; doi:10.3389/fneur.2025.1549091)
Supplement: Supplementary file 1 [file Table_1.docx]

Table S1. Summary of the limits 5 and 95% confidence interval of each retinal layer thickness difference between the control group and the multiple sclerosis subgroups.

| Retinal Layer | MS subgroup | Central | Inner Inferior | Inner outter | Nasal inferior | Nasal outter | Superior inner | Superior outter | Temporal inferior | Temporal outter |
| --- | --- | --- | --- | --- | --- | --- | --- | --- | --- | --- |
| NFL | PP | -0.03, 1.69 | -5.26, -2.55 | -10.20, -5.13 | -3.65, -1.44 | -13.63, -7.29 | -4.47, -1.89 | -7.48, -2.95 | -1.01, 0.50 | -1.68 -0.17 |
|  | RR | -0.32, 0.33 | -2.52, -1.51 | -4.03, -2.13 | -0.96, -0.14 | -5.76, -3.39 | -1.66, -0.70 | -3.76, -2.07 | -0.17, 0.40 | -0.46 0.11 |
|  | SP | -0.12, 0.69 | -3.94, -2.66 | -7.26, -4.86 | -1.70, -0.66 | -10.32, -7.32 | -3.25, -2.03 | -5.95, -3.81 | -0.34, 0.39 | -0.67 0.06 |
| GCL | PP | -4.56, -0.73 | -11.12, -5.59 | -2.25, -0.28 | -13.14, -7.00 | -5.81, -2.66 | -11.48, -5.79 | -3.10, -0.84 | -11.35, -5.59 | -4.72 -1.76 |
|  | RR | -1.71, -0.27 | -6.51, -4.45 | -1.58, -0.83 | -7.35, -5.06 | -3.98, -2.80 | -6.92, -4.80 | -2.12, -1.28 | -6.13, -3.98 | -2.88 -1.78 |
|  | SP | -4.26, -2.45 | -12.68, -10.06 | -3.01, -2.06 | -14.70, -11.80 | -6.66, -5.15 | -13.19, -10.51 | -4.02, -2.95 | -12.19, -9.47 | -5.12 -3.73 |
| IPL | PP | -2.76, 0.41 | -4.34, -1.48 | -2.10, 0.22 | -4.17, -1.02 | -3.49, -0.71 | -3.95, -1.07 | -1.98, 0.39 | -6.23, 0.44 | -3.24 -0.57 |
|  | RR | -1.01, 0.18 | -1.82, -0.75 | -1.31, -0.43 | -1.79, -0.62 | -1.32, -0.27 | -1.96, -0.88 | -1.14, -0.25 | -2.32, 0.16 | -1.67 -0.66 |
|  | SP | -1.94, -0.43 | -4.58, -3.23 | -3.04, -1.92 | -4.48, -2.99 | -3.39, -2.05 | -4.34, -2.99 | -2.72, -1.57 | -5.53, -2.37 | -3.52 -2.23 |
| INL | PP | -3.39, 0.24 | -1.63, 0.64 | -0.53, 1.51 | -1.78, 0.82 | -1.58, 0.79 | -1.23, 1.08 | -0.81, 1.28 | -1.31, 0.98 | -1.56 0.60 |
|  | RR | -0.36, 1.00 | 0.37, 1.22 | -0.31, 0.46 | 0.21, 1.17 | -0.81, 0.08 | 0.08, 0.94 | -0.43, 0.36 | 0.17, 1.03 | -0.28 0.54 |
|  | SP | -1.79, -0.04 | -1.44, -0.37 | -1.48, -0.49 | -1.34, -0.11 | -2.54, -1.39 | -1.43, -0.34 | -1.56, -0.55 | -1.25, -0.17 | -1.53 -0.48 |

For abbreviations refer to Table 2 and Figure 2 legends.
